# Identification of Three Novel Splicing Variants and Expression Analysis of Chicken GPR1 Gene

**DOI:** 10.1155/2017/1074054

**Published:** 2017-01-22

**Authors:** Xueyou Zhang, Qihai Xiao, Kai Tian, Yan Wang, Xiaoling Zhao, Huadong Yin, Diyan Li, Qing Zhu

**Affiliations:** Institute of Animal Genetics and Breeding, Sichuan Agricultural University, Chengdu, Sichuan 611130, China

## Abstract

*GPR1* is a G protein-coupled receptor that plays critical roles in eukaryotic cells: typically, response to glucose stimulation, lipid accumulation, and transmitting nutrition signals to cAMP pathway. However, the alternative splicing of the* GPR1 *gene and its expression pattern in chicken tissues and ovarian follicles were unknown. In our current study, we used RACE-PCR to identify three* GPR1 *variants, including the full-length variant (*GPR1-va1*) and two alternatively spliced variants (*GPR1-va2*,* GPR1-vb*). Quantitative real-time PCR examined the expression pattern of* GPR1 *mRNA in chicken tissues and ovarian follicles. The result reveals that the coding sequence of the three variants cDNA is 1053, 1053, and 627 bp in length, encoding 350, 350, and 208 amino acids, respectively. The three variants of* GPR1 *show similar tissue distributions;* GPR1 *expression was abundant in the abdominal fat, lung, and heart. With the follicular development, the expression of* GPR1* gene gradually increased, and* GPR1-va1 *and* GPR1-va2 *spliced variants expression in F2 were significantly higher than in F5, F4, and prehierarchical follicles (*P* < 0.05). Taken together, we found three novel variants of* GPR1*, and the results of* GPR1* expression profiling in adipose tissues and ovarian follicles suggest that* GPR1* may play a significant role in the lipid accumulation and progression of follicular development.

## 1. Introduction

Many signaling transductions are mediated by G protein-coupled receptors (GPCRs) in eukaryon [1]. G protein-coupled receptors exist in eukaryotes, including yeast, choanoflagellates, and animals [2].* GPR1* is a G protein-coupled receptor (GPCR), originally found in human [3], which was identified by in vitro experiment as receptor for chemerin [4, 5].* GPR1* and chemerin are related to adipogenesis [6–9], circadian appetite regulation [10], cell chemotaxis [11], inflammation [6, 12, 13], and phosphorylation of ERK and Akt [14].

Alternative splicing (AS) of pre-mRNA can generate diversity form protein subtypes from a single gene [15–17]. In many instances, coding sequence was affected by alternative splicing, which would result in the production of diverse proteins [18]. Various proteins would be produced due to different open reading frames [19]. In some kind of situation, partly different proteins may have various functions, lacking or having a special function [20]. Recent studies using next generation sequencing have demonstrated that AS could generate huge transcriptional isoforms of mammalian gene [16, 21–23]. Alternative splicing has been demonstrated to act as a major mechanism that modulates gene expression and function of GPCRs [24–26].

In this study, we identified three novel* GPR1 *splice variants. We designated the novel variants* GPR1-va1*,* GPR1-va2, *and* GPR1-vb*.* GPR1-va1* and* GPR1-va2 *and* GPR1-vb *use the same translation start codon. However, the CDS of* GPR1-vb *was different from* GPR1-va1* and* GPR1-va2*, and thus as a result they have different amino acid sequences. We examined the different expression profiling between the three variants in tissue and ovary follicles distribution using qRT-PCR. These data could increase our knowledge of* GPR1 *mRNA diversity and provides the basis for further functional research.

## 2. Materials and Methods

### 2.1. Experimental Animals and Tissue Sampling

Three producing female Lohmann pink tissues (*Gallus gallus*) in the fiftieth week were selected for sampling from the Experimental Farm for Fowl Breeding at Sichuan Agricultural University (Sichuan, China). These chickens were hatched on the same day and grown under the same natural conditions of light and temperature. Eye, brain, hypothalamus, pituitary, ovary, oviduct, adipose tissues, muscle tissues, POF, lung, spleen, kidney, and ovarian and granulosa cells from 1 to 2, 2 to 3, 3 to 4, 4 to 5, 5 to 6, 6 to 7, 7 to 8, and 8 to 9 mm diameter prehierarchical follicles, and F5–F1 (measuring F1 > F2 > F3 > F4 > F5 in diameter) hierarchical follicles were collected, rapidly frozen in liquid nitrogen, and finally stored at −80°C until RNA extraction. The protocol for bird treatment was in accordance with the Sichuan Agricultural University Council on Animal Care Guidelines.

### 2.2. Reverse Transcription PCR

Total RNA was extracted from these samples with TRIzol (TaKaRa, Dalian, China). cDNAs were synthesized using a PrimeScript® RT Reagent Kit (TaKaRa, Dalian, China) according to the manufacturer's instructions. In brief, step 1: the 10.0 *μ*L reaction consisted of 1.0 *μ*L of total RNA, 2.0 *μ*L of 5x gDNA Eraser Buffer, 1.0 *μ*L of gDNA Eraser, and 5.0 *μ*L of RNase-Free dH_2_O. Thermal cycling was executed for 2 min at 42°C. Step  2: the 20.0 *μ*L reaction consisted of 10 *μ*L the reaction solution from step 1, 1.0 *μ*L of PrimeScript RT Enzyme Mix I, 1.0 *μ*L of RT Primer Mix, 4.0 *μ*L of 5x PrimeScript Buffer 2 (for Real Time), and 4.0 *μ*L of RNase-Free dH2O. Thermal cycling was executed for 15 min at 37°C and then 5 sec at 85°C.

### 2.3. 5′-/3′-Rapid Amplification of cDNA Ends-PCR

Total RNA was extracted from mix sample (hypothalamus, pituitary, oviduct, adipose tissues, and muscle tissues) with RNeasy Mini Kit (Qiagen, German) and subsequently processed with the SMART-rapid amplification of cDNA ends (RACE) cDNA Amplification Kit (Clontech, USA). RACE-PCR were carried out using 1.5 *μ*L of 5-fold diluted 3′-RACE (or 5′-RACE)-ready cDNA as template in a 50 *μ*L under the following cycling conditions: The first-round PCR: 94°C for 3 min; 5 cycles at 94°C for 30 s and 72°C for 4 min; 5 cycles at 94°C for 30 s, 70°C for 30 s, and 72°C for 4 min; 25 cycles at 94°C for 30 s, 68°C for 30 s, and 72°C for 4 min; the second-round PCR: 94°C for 3 min and 16 cycles at 94°C for 30 s, 68°C for 30 s, and 72°C for 4 min; the third-round PCR: 94°C for 3 min and 25 cycles at 94°C for 30 s, 68°C for 30 s, and 72°C for 4 min. Detailed information on the RACE-PCR primers (1.1–3.3) is provided in Table 1.

### 2.4. Quantitative Real-Time PCR

Total RNA was isolated from these samples of each hen with TRIzol reagent (TaKaRa, Dalian, China). Approximately 1 *μ*g of DNase-treated RNA from each sample was reverse transcribed with a cDNA Synthesis Kit. The cDNA samples were diluted 4-fold and subjected to qRT-PCR on a C1000™ Thermal Cycler (Bio-Rad, CA, USA). Each qRT-PCR was performed in a 25 *μ*L volume containing 1.5 *μ*L of diluted cDNA, 12.5 *μ*L of 2x SYBR Premix Ex-Taq II (TaKaRa, Dalian, China), and 1.2 *μ*L of variant-specific primer pair mix (10 pmol/*μ*L each primer). All variant-specific primer pairs were run with the same cycling conditions: 95°C for 30 s followed by 46 cycles of 95°C for 5 s and 60°C for 30 s with a final melting curve analysis (from 65°C to 95°C at a rate of 0.5°C per 5 s). The melting curve analyses showed that the amplification efficiency of each variant-specific primer pair was higher than 97%. Negative and positive controls were included in each experiment as quality control and threshold cycle (Ct) calibration steps.

The expression levels of the target genes were calculated using geNORM algorithms [27] based on the geometric means of two reference genes: *β*-actin and GAPDH. Each sample was run in triplicate.

Detailed information on the qRT-PCR primers (4.1–7.2) is provided in Table 1.

### 2.5. Cloning and Sequencing of PCR Products

Following amplification, RACE-PCR products were purified with TaKaRa MiniBEST DNA Fragment Purification Kit Ver 4.0 (TaKaRa, Dalian, China). The purified PCR products were performed in a 50 *μ*L volume containing 30 *μ*L of cDNA and 0.25 *μ*L of Ex-Taq (TaKaRa, Dalian, China) under the following cycling conditions: 94°C for 3 min and 30 cycles at 98°C for 10 s and 68°C for 4 min and 72°C for 5 min. Following amplification, products were purified with TaKaRa MiniBEST DNA Fragment Purification Kit Ver 4.0 (TaKaRa, Dalian, China). The purified PCR products were ligated into a pMD-19 T vector (TaKaRa, Dalian, China). Positive clones were selected by sequencing, which was performed by the Shanghai Invitrogen Biology Company. Finally, the T-A clone products of* GPR1 *were directly sequenced by the Chengdu Tsingke Biological Engineering Technology.

### 2.6. Sequence Analysis

All primers were designed using Primer Premier 5.0 software and synthesized by Chengdu Tsingke Biology Company. cDNA and DNA segments obtained from sequencing were edited, assembled, and aligned with Editseq, Seqman and MegAlign, respectively, in Lasergene 7.1 software (DNASTAR, Madison, WI, USA). The transmembrane helices were predicted by TMHMM (http://www.cbs.dtu.dk/services/TMHMM-2.0). The* GPR1* sequences of other vertebrates (retrieved from GenBank) were aligned using ClustalW software (version 1.7; DDBJ). The phylogenetic tree constructed from the alignment was generated with the neighbor-joining method using Molecular Evolutionary Genetic Analysis (MEGA) software version 5.1 (http://www.megasoftware.net/), followed by phylogeny tests with 1000-bootstrap replicates. Spidey (http://www.ncbi.nlm.nih.gov/IEB/Research/Ostell/Spidey/) was used to analyze AS patterns. Open reading frames (ORFs) and translated proteins were predicted using the ORF Finder in NCBI. Proteins 3D structures were created using the I-TASSER server (http://zhanglab.ccmb.med.umich.edu/I-TASSER/) [28–30] and Rosetta server (http://robetta.bakerlab.org/) [31] and visualized using PyMOL [32].

### 2.7. Statistical Analysis

All data were analyzed by a one-way analysis of variance (ANOVA), which was followed by Duncan's multiple range test, using the SAS 9.0 statistical software for Windows (SAS Institute Inc., USA). Values were expressed as the mean ± SEM, *n* = 3. Differences were considered significant at *P* < 0.05.

## 3. Results

### 3.1. Analysis of* GPR1* Variants Sequence Characteristics

#### 3.1.1. Multiple* GPR1* Variants

**Figure 1:**
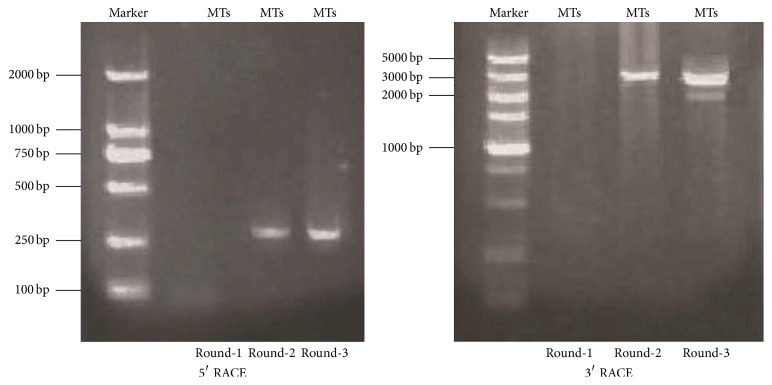
Gel electrophoresis images of* GPR1 *RACE-PCR products. PCR products were amplified with nested PCR. Gel pictures analysis suggesting the presence of multiple* GPR1* variants. Note a single band in 5′-RACE-PCR and multiple amplicons in 3′-RACE-PCR. The PCR products of GPR1 were separated on 1% agarose gel following electrophoresis and visualized with ethidium bromide. “MTs” represents mixtures of cDNA (hypothalamus, pituitary, oviduct, adipose tissues, and muscle tissues). Round 1, Round 2, and Round 3 represent the first-round PCR, the second-round PCR, and the third-round PCR, respectively.

To investigate chicken* GPR1*, we cloned splice variants of* GPR1 *by RACE-PCR. We obtained three full-length mRNA sequences alternative splice variants (Figure 1). Similarity analysis identified three* GPR1* variants:* GPR1-va1* (KX156840),* GPR1-va2 *(KX156841), and* GPR1-vb *(KX156842).

BLASTn alignments showed that although all variants are most similar to vertebrate* GPR1*,* GPR1-va1* shows the highest similarity to* GPR1 *mRNA, with 99%, 94%, 93%, and 86% similarity to* Meleagris gallopavo*,* Anser cygnoides*,* Anas platyrhynchos,* and* Coturnix Japonica*, respectively. In addition, MegAlign analysis suggests that they are all splice variants.

**Figure 2:**
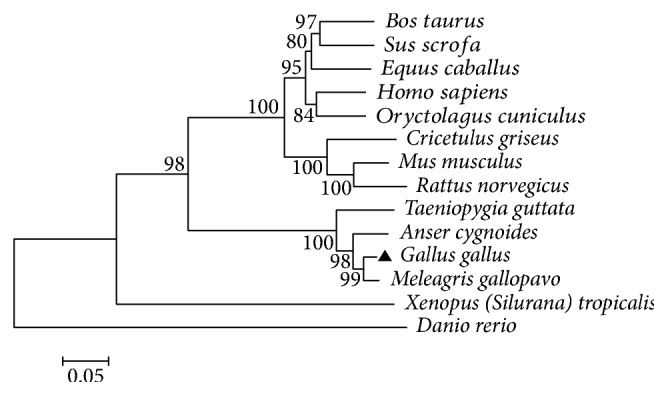
The phylogenetic tree of* GPR1* sequence from different vertebrate species. Neighbor-joining analysis based on the Poisson correction model with 1000-bootstrap replicates was performed using MEGA 5.1 software. Numbers at each branch indicate the percentage of times a node was supported in 1000-bootstrap replicates. The species names and GenBank accession numbers of the* GPR1 *sequences shown are as follows:* Anser cygnoides* (XM_013171695.1),* Bos Taurus* (XM_005202718.3),* Cricetulus griseus* (XM_003502119.1),* Danio rerio* (XM_001343478.5),* Equus caballus* (XM_014732609.1),* Gallus gallus* (XM_004942654.2),* Homo sapiens* (NM_001261453.1),* Meleagris gallopavo* (XM_010713490.1),* Mus musculus* (XM_011238518.1),* Oryctolagus cuniculus* (XM_008259021.1),* Rattus norvegicus* (XM_008767077.1),* Sus scrofa* (XM_013984415.1),* Taeniopygia guttata* (XM_012574956.1), and* Xenopus (Silurana) tropicalis* (XM_004917751.2).

Phylogenetic tree analysis using* GPR1-va1 *sequences from other vertebrate species has shown that the chicken* GPR1-va1* is most closely related to the* GPR1* sequence in* Meleagris gallopavo* followed by those in* Anser cygnoides* and* Taeniopygia guttata* (Figure 2).

#### 3.1.2. Structural Analysis of* GPR1* Variants

Spidey analysis revealed that* GPR1* comprises two exons and one intron (Figure 4(a)). All three* GPR1* variants are generated from a single sequence through different splicing modes (Figure 4(b)). In addition, all splicing modes are consistent with the canonical 5′-GU—AG 3′-donor—acceptor splice site pairs rule. ORF Finder and Spidey analysis of the three* GPR1* variants showed that although each variant has an identical short 5′-UTR (untranslated region), the CDS and 3′-UTRs vary significantly in size, ranging from 627 to 1053 bp and 1139 to 1634 bp, respectively (Figure 4(b) and Table 2).

**Figure 3:**
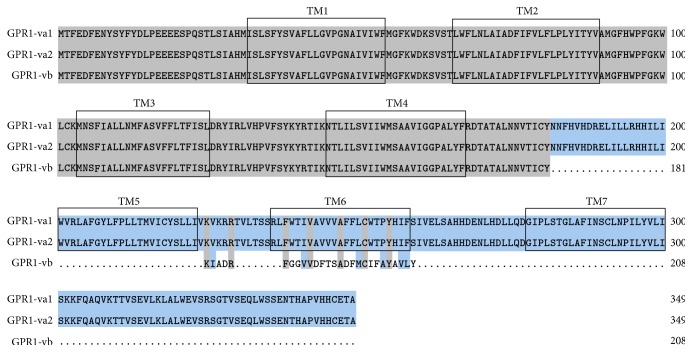
Amino acid sequence alignment of GPR1-va1, GPR1-va2, and GPR1-vb. Sequences of GPR1-va1, GPR1-va2, and GPR1-vb were aligned by DNAMAN. Putative transmembrane domains were shaded in grey.

**Figure 4:**
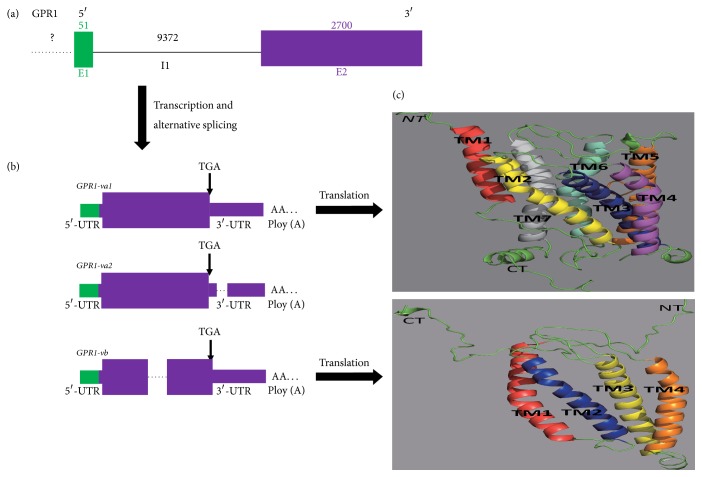
Structure analysis of* GPR1*. (a) Structure analysis of the* GPR1* genomic locus (*GPR1*). Exons were denoted by thick boxes marked “E1, E2” with different colors. Introns were denoted as solid lines and annotated I1. Unknown region was marked “$\underset{..}{?}$”; the size of an exon or intron was indicated by a number above the exon or intron. (b) Structure analysis of* GPR1 *variants (*GPR1-va1*,* GPR1*-*va2 *and* GPR1-vb*). The splicing model was labeled as 3′-ATSS (E2) (3′-alternative tailing site selection in exon 2) and intron retention in exon 2. The UTR (untranslated region) and poly(A) (polyadenylation site) of each variant were indicated by thin colored boxes and “AA…”, respectively. CDS (coding sequence) was denoted by a thick colored box;* GPR1-va1 *and* GPR1-va2* have the same coding sequence. (c) Transmembrane domain prediction of putative proteins encoded by three* GPR1 *variants.* GPR1-va1* and* GPR1-va2* exhibited typical type GPCR features consisting of an extracellular N-terminus, 3 intracellular and extracellular loops, and 7 transmembrane a-helical domains.* GPR1-vb* was truncated protein, with 4 transmembrane domains. Detailed information for (c) is shown in Figure 3.

I-TASSER (Figure 4(c)), Rosetta (supplementary Fig. 1B, in Supplementary Material available online at https://doi.org/10.1155/2017/1074054), TMHMM, and DNAMAN (Figure 3) comparison of the putative* GPR1* receptors encoded by these variants revealed that* GPR1 *exhibits the typical type A GPCR features including seven transmembrane a-helical domains connected by three extracellular and three intracellular loops. However,* GPR1-vb* variants were all truncated proteins, with partial transmembrane domains from type A GPCRs.* GPR1-vb* spanned 627 bp and encoded 208 amino acids, which share 90.4% similarity with* GPR1-va* (*GPR1-va1 *and* GPR1-va2* contain the same amino acid sequence).

### 3.2. Expression Analysis of* GPR1* Variants mRNA in Lohmann Pink Tissues

**Figure 5:**
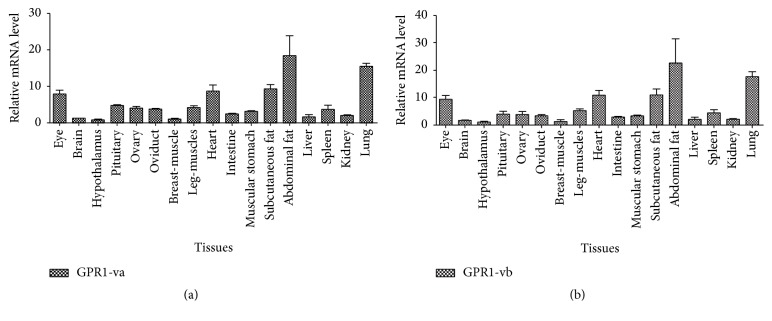
Tissue distribution of* GPR1 *splice variants. Expression profiling of* GPR1* in the Lohmann pink tissues. The relative levels of expression for* GPR1* were calculated relative to* GAPDH *and *β-actin *using 2^−ΔΔCt^ method. Values are mean ± SEM, *n* = 3. The significance of differences in the levels of expression of* GPR1 *mRNA was determined by ANOVA. Means with the same letter are not significantly different (*P* < 0.05).

qRT-PCR analysis showed* GPR1 *variants mRNA expression in all tissues (Figure 5). The highest expression level of* GPR1-va* (*GPR1-va1* and* GPR1-va2* have the same coding sequence) in the Lohmann pink tissues was detected in the abdominal fat and lung (*P* < 0.05).* GPR1-vb* mRNA expression in the abdominal fat was also significantly high compared to other tissues (*P* < 0.05). In contrast, the lowest expression level was observed in the hypothalamus (*P* < 0.05).

**Figure 6:**
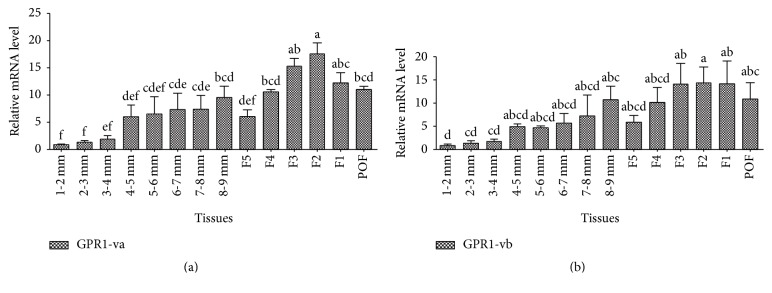
Expression profiling of* GPR1 *splicing variants in the hierarchical and prehierarchical follicles of Lohmann pink ovary. The relative levels of expression for* GPR1* were calculated relative to* GAPDH* and*β-actin* using 2^−ΔΔCt^ method. Values are mean ± SEM, *n* = 3. The significance of differences in the levels of expression of* GPR1 *mRNA was determined by ANOVA. Means with the same letter are not significantly different (*P* < 0.05).

qRT-PCR revealed* GPR1 *variants expression in different follicles of the Lohmann pink ovary (Figure 6).* GPR1* transcripts were detected in all experimental hierarchical follicles and prehierarchical follicles of the Lohmann pink ovary. With follicular development,* GPR1-va* expression levels gradually increased, with levels in F2 significantly higher than that in F4, F5, and prehierarchical follicles (*P* < 0.05). There was also a high expression of* GPR1-vb* in the F2 compared to 1 to 2, 2 to 3, and 3 to 4 mm diameter prehierarchical follicles (*P* < 0.05). The lowest* GPR1-va* and* GPR1-vb* gene expression were detected in 1 to 2 mm diameter prehierarchical follicles (1-2 mm).

## 4. Discussion

Previous studies on* GPR1* mainly used rodent animal models and little was known about the molecular characteristics in aves such as chicken. In this study, we identified three novel* GPR1* splice variants that have partially different CDS and 3′-UTR from the* GPR1* originally reported.* GPR1-va1*,* GPR1-va2, *and* GPR1-vb* are alternatively spliced variants. mRNA for* GPR1-va1* uses exon 1 and exon 2, mRNA for* GPR1-va2 *lacks a segment sequence in 3′-UTR (Figure 4(b)), and mRNA for* GPR1-vb* lacks a segment sequence in CDS (Figure 4(b)). Interestingly, we compared the forecasting method of I-TASSER and Rosetta, and we find some differences in 3D structure (Figure 4(c) and supplementary Fig. 1B). Perhaps the main reason is that I-TASSER and Rosetta adopted TBM+FM and FM forecasting method, respectively [33]. The differently three-dimensional structure of the protein determines the different function of protein; therefore, improving the performance of protein structure prediction algorithm is a key technique in the further study. To date, many* GPCRs *splice variants have been reported invertebrates [26, 34–36]. Alternative splicing (AS) that generates complexity before mRNA can produce distinct mRNA and protein isoforms [37, 38]. AS could result in physiological diversity such as differences in tissue distribution, ligand-binding properties, signaling pathways, and coupling efficiency with G*α* protein [39]. The tissues distributed of* GPR1-va* (*GPR1-va1* and* GPR1-va2*) is similar with* GPR1-vb*. But* GPR1-va *was more abundantly expressed (Figures 5 and 6).

Because* GPR1-va1*,* GPR1-va2,* and* GPR-vb* use the same transcription initiation site, the ratio of mRNAs for* GPR1-va1*,* GPR1-va2, *and* GPR-vb* may be determined by posttranscriptional regulation, such as splicing efficiency and mRNA stability [40, 41]. The stability of the mRNA may be different between* GPR1-va1*,* GPR1-va2,* and* GPR-vb*. Splicing is regulated by several factors such as splice site recognition, splicing regulators, and RNA secondary structure [42–44]. In vertebrate genes, splice sites are not well conserved, which allows alternative splicing to occur frequently [45]. If introns are retained within the CDS, the site of a stop codon would contribute to the production of a truncated peptide (if inserted close to the 3′-end) or the absence of a protein product (if inserted downstream of the start codon) [46]. In our study, the stop codon of* GPR1-vb* was not changed relative to* GPR1-va1* and* GPR1-va2*; we found that the translatability of about 426 nucleotides might indeed be interrupted by the retention of an intron in the CDS. This type of event was typically disregarded owing to the absence of protein products; however, intron retention might also contribute to the diversification of the information carried by genes, by producing functional RNA [47].

*GPR1 *is ubiquitously expressed in most tissues, and* GPR1* expression profile is the same as swine [6] and mouse [10, 48]. In our study,* GPR1-va* and* GPR-vb* mRNA were expressed in all tissues examined and in a highly tissue-specific manner in the Lohman pink tissues. The high levels of* GPR1-va* and* GPR1-vb *were detected in the adipose tissue which implicates its potential key role in regulating chicken adipocyte development. In addition,* GPR1-va* and* GPR1-vb *mRNA were expressed at the highest levels in the abdominal fat, followed by muscle, lung, subcutaneous fat, heart, eye, and other tissues, which confirmed* GPR1 *mRNA tissue-specific expression in different chicken tissues. This result is coincident with previous studies in mouse (gender and age not indicated, obese mice) [10, 49]. However, the highest* GPR1 *mRNA expression level is found in the kidney of pig (males, 2.5 months old) [6] and in the skeletal muscle of mouse (gender and age not indicated, obese/diabetic mice) [50]. Therefore, we cannot eliminate the possibility that* GPR1-va1*/*GPR1-va2*/*GPR1-vb* shows different expression patterns in species-, gender-, or temporal-specific profiles or that it shows different functions within the adipose tissue.

In our current study,* GPR1-va1*,* GPR1-va2,* and* GPR-vb* expression gradually increased with follicular development, suggesting that* GPR1-va1*,* GPR1-va2,* and* GPR-vb *may regulate follicular development in the Lohman pink tissues. However, the level of* GPR1-va1*,* GPR1-va2,* and* GPR-vb *expression was highest in the F2 than in the other follicles (prehierarchical follicles, F5, F4, F3, F1, and POF). Previous studies showed that the high level of* GPR1* was detected in subcutaneous fat [6], which speculated that* GPR1* could play a role in lipid accumulation. Moreover, lipid and lipid metabolism play a crucial role in cell survival and proliferation [51, 52]. In addition,* GPR1* and* CMKLR1* are the coreceptors for chemerin, and they have the closest phylogenetic relationship in the family of chemoattractant receptors [6, 53]. Goralski et al. study suggested that chemerin and* CMKLR1* could regulate adipogenesis and adipocyte metabolism through ERK1/2 signaling pathway [54]. Various follicle classes have different lipid characteristics [55]; by adding* GPR1* antibody and PI3K signaling inhibitor we find that the chemerin/GPR1 and PI3K signaling pathways may be involved in follicular development [56]. Therefore,* GPR1* is the only known receptor for chemerin which also may regulate the follicular development through regulating follicular lipid accumulation. In this study, expression of* GPR1* showed an increased tendency gradually with follicular growth; however, the expression of* GPR1* tends to decrease in the F1, suggesting that follicular internal environment may be changed such as lipid metabolism. Interestingly, study on* GPR1* KO mice speculated that* CMKLR1* compensated for the loss of* GPR1* function [10]. As such future studies examine follicular development and lipid metabolism through both* CMKLR1* and* GPR1* when investigating the signal transduction mechanisms of chemerin function.

## 5. Conclusion

In this study, we cloned three alternative splice variants of* GPR1 *full-length mRNA sequences from Lohmann pink tissues. The* GPR1* transcript was widely distributed in various tissues. With follicular development,* GPR1* gene expression gradually increased;* GPR1-va* expression in F2 was significantly higher than in F5. The results of the* GPR1 *expression profiling of ovarian follicles suggested that* GPR1 *plays key role in follicular development through regulating the lipid levels. Therefore, our findings increase our knowledge of* GPR1* mRNA diversity and provide a solid basis for further molecular mechanism research.

## Supplementary Material

I-TASSER and Rosseta to predict the 3D structure of GPR1

## Figures and Tables

**Table 1:** Primers used to study GPR1.

S. number	Primer name	Sequence (5′- to 3′-end)	Source	Purpose
1.1	GR1-5′-1	CAGATGACAATGGCATTGCCTGGGACTC	Conserved domains of published sequences (XM_015289515.1, 971405019)	To obtain 5′-UTR and 3′-UTR
1.2	GR1-5′-2	TATGGGCAATGCTGAGGGTGGACTGG		
1.3	GR1-5′-3	CTTCCTCAGGCAGGTCATAGAAATAAGAGTAG		
2.1	UPM-long	ATAGGGCAAGCAGTGGTATCAACGCAGAGT		
	UPM-short	ATAGGGC		
3.1	GR1-3′-1	GAGGAAGGCAGTAGCCAAGTGAAGCAGC		
3.2	GR1-3′-2	CACACTGAAGTCACTCTGAACTCTACAGATCACT		
3.3	GR1-3′-3	GCTACAGAGAGCACATCCCTGACTTACAGTGT		

4.1	GPR1-va-F	TGACTGTTCATCACCATCCATCT		To measure expression levels of GPR1-va and GPR1-vb by qRT-PCR, respectively
4.2	GPR1-va-R	ATGCTGCAACCGCACCAC		
5.1	GPR1-vb-F	TGTCACCATTTGCTACAAAATAGC		
5.2	GPR1-vb-R	ATGCTGCAACCGCACCAC		

6.1	*β*-Actin-F2	TGTGCTGTCCCTGTATGCCTC	Specific regions of *Gallus gallusβ*-actin (L08165.1) Specific regions of *Gallus gallus* GAPDH (NM_204305.1)	
6.2	*β*-Actin-R2	GGAGGGCGTAGCCTTCATAGA		
7.1	GAPDH-F	CCAGAACATCATCCCAGCGTC		
7.2	GAPDH-R	ACGGCAGGTCAGGTCAACAA		

**Table 2:** Sequence analysis of three *GPR1* variants.

Isoform	Total length (nt)	5′-UTR length (nt)	CDS length (nt)	3′-UTR length (nt)	Poly(A) length (nt)
GPR1-va1	2875	160	1053	1634	28
GPR1-va2	2377	160	1053	1139	25
GPR1-vb	2343	160	627	1528	28
