# Supplementary material for: Identification of Three Novel Splicing Variants and Expression Analysis of Chicken GPR1 Gene
Source: Biomed Res Int. 2017 Jan 22;2017:1074054. doi: 10.1155/2017/1074054 (PMC5292125; doi:10.1155/2017/1074054)
Supplement: Supplementary file 1 — I-TASSER and Rosseta to predict the 3D structure of GPR1 [file 1074054.f1.pdf]

Supplementary figure

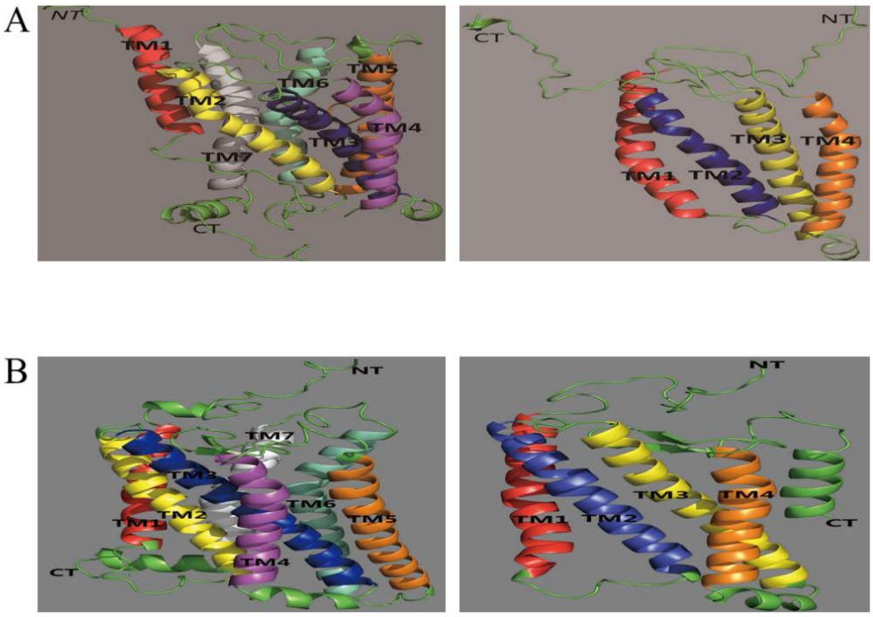

Supplementary figure.1 I-TASSER and Rosseta to predict the 3D structure of GPR1. The 7 transmembrane domain of proteins encoded by *GPR1-va* variants, and the 4 transmembrane domain of proteins encoded by *GPR1-vb* variants. (A) I-TASSER to predict the structure of GPR1. (B) Rosetta to predict the structure of GPR1.
